# Identification of Stem Rust Resistance Genes in the Winter Wheat Collection from Southern Russia

**DOI:** 10.3390/plants8120559

**Published:** 2019-11-30

**Authors:** Andrey V. Alabushev, Nataliya N. Vozhzhova, Natiya T. Kupreyshvili, Nikolay V. Shishkin, Dmitry M. Marchenko, Elena V. Ionova

**Affiliations:** 1 Federal State Budgetary Scientific Institution “Agricultural Research Center “Donskoy” (FSBSI “ARC “Donskoy”), 347740 Zernograd, Rostov Region, Russia; 2 Laboratory of Marker Breeding FSBSI “ARC “Donskoy”, 347740 Zernograd, Rostov Region, Russia; 3 Laboratory of Plant Immunity and Protection FSBSI “ARC “Donskoy”, 347740 Zernograd, Rostov Region, Russia; 4 Department of Winter Wheat Breeding and Seed-Growing FSBSI “ARC “Donskoy”, 347740 Zernograd, Rostov Region, Russia; 5 Laboratory of Plant Physiology FSBSI “ARC “Donskoy”, 347740 Zernograd, Rostov Region, Russia

**Keywords:** MAS, *Sr2*, *Sr31*, *Sr38*, *Sr44*, stem rust

## Abstract

The high yield potential of winter wheats cannot be realized due to disease pressure under field conditions. One of the most harmful of such diseases is stem rust, hence the constant search for sources of resistance and the development of new varieties resistant to stem rust is of great relevance. This study deals with the identification of stem rust resistance genes in a collection of winter wheats grown in Southern Russia. This genepool has not been studied yet. A total of 620 samples of winter soft wheat from various ecological and geographical zones were tested under field conditions. To identify the specific genes or alleles responsible for resistance, all samples were genotyped using PCR. As a result, the groups of resistant samples, carrying the *Sr2*, *Sr31*, *Sr38* and *Sr44* genes in various combinations, were identified. Most of the stem rust resistance was provided by the presence of the effective *Sr44* gene. This information can be used in the future breeding work for stem rust resistance.

## 1. Introduction

Winter wheat is one of the main sources of food for the population in most countries of the world [1]. It is necessary to obtain stable and high grain yields in order to provide the population with food. The varieties developed by breeders have a high productive potential, which cannot fully be realized because of crop diseases [2].

One of the most harmful winter wheat diseases is stem rust (*Puccinia graminis f. sp. tritici*). The disease appears after flowering on stems and leaf sheaths, in the form of rust-brown oblong powdering with urediniospore pustules that coalesce in the form of brown stripes and tearing of the epidermis. This disease can reduce winter wheat yields by up to 80% [3]. The Ug99 stem rust emergence in 1999 [4], which overcame the rust resistance of many varieties [5], made it urgent to find sources of resistance and to identify new winter wheat varieties with these genes.

The stem rust resistance genes mostly used in the world breeding are the *Sr31* (located in the translocation with the *Lr26*, *Yr9* and *Pm8*) and *Sr38* genes (linked to the gene for adult plant resistance to leaf rust *Lr37* and *Yr17*) [6]. Currently, Ug99 overcomes their resistance. According to Terefe [7] there was virulence to the main stem rust resistance genes *Sr5*, *Sr6*, *Sr9e* and *Sr38* of the TTKSF race, which is one of the variations of the highly virulent race Ug99. This race was found in Africa, and it was Boshoff who reported stem rust isolates virulent to Sr38 for the first time [8].

Sibikeev [9] reported the detection of stem rust isolates overcoming the *Sr31* gene resistance in the central non-black-earth zone of Russia in the years of 2012 and 2013. However, the *Sr31* and *Sr38* genes keep providing resistance to local stem rust races in some regions of the world [10,11,12]. In China, the *Sr38* and *Sr44* genes and their combination with the *Sr25* and *Sr2* genes proved to be effective stem rust resistance genes [13]. The *Sr38*, *Sr26* and *Sr36* genes remain effective against the Indian race of stem rust [11]. In the south of Russia, in the Southern Federal District, gene *Sr31* keeps maintaining its effectiveness [14,15].

The *Sr2* and *Sr44* genes belong to the group of effective genes, resistant to the Ug99 race and its other phenotypes [16]. The *Sr2* gene provides partial but long-lasting stem rust resistance for more than 50 years [17,18]. The gene is non-specific and effective against many Puccinia graminis tritici isolates in all wheat-growing regions of the world. [19]. The stem rust resistance gene *Sr44* is resistant to the complex Ug99 race, namely TTKSK, TTSKT and TTTSK [20].

We have not studied the genotypes of the resistant winter soft wheat samples collected in our research center before. The purpose of our work was to identify the sources of stem rust resistance genes in the winter soft wheat collection samples of the Federal State Budgetary Scientific Institution “Agricultural Research Centre “Donskoy” (FSBSI “ARC “Donskoy”) for their further use in breeding.

## 2. Results

The PCR analysis showed a wide variety of samples according to the presence of the studied stem rust resistance genes. For PCR analysis, we used all 620 lines.

**Figure 1:**
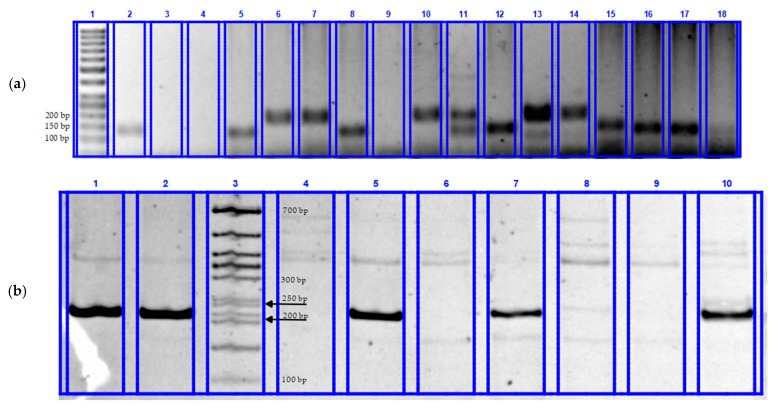

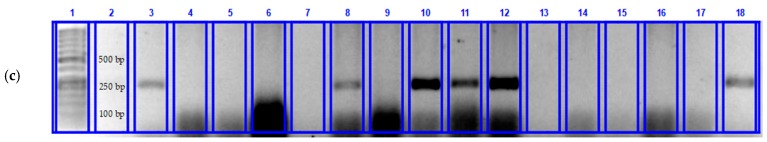
Electrophoregrams for screening winter soft wheat samples by determining stem rust resistance genes: (**a**)—*Sr2*, agarose gel: 1—GeneRuler 50 + bp (ThermoScientific), 2—WIR64679 (positive control of experience), 3—H_2_O deionized (negative control of experience), 4—WIR42910, 5—K15-0542, 6—K15-0553, 7—K15-0580, 8—K16-0147, 9—K16-0148, 10—K16-0149, 11—K16-0150, 12—K17-0278, 13—K17-0283, 14—K17-0287, 15—K17-0292, 16—K17-0294, 17—K17-0300, 18—K17-0302; (**b**)—*Sr31*, PAGE: 1—TchLr26 (positive control of experience), 2—K17-0216, 3—The molecular weight marker Evrogen 50 + bp (50–700 bp), 4—K17-0218, 5—K17-0224, 6—K17-0223, 7—K17-0232, 8—K17-0217, 9—K17-0219, 10—17-0256; (**c**)—*Sr38*, agarose gel: 1—GeneRuler 50 + bp (ThermoScientific), 2—H_2_O deionized (negative control of experience), 3—TchLr37 (positive control of experience), 4—WIR42910, 5—WIR53496, 6—WIR56750, 7—WIR56753, 8—K15-0672, 9—K15-0588, 10—K15-0604, 11—K15-0605, 12—K15-0606, 13—K15-0680, 14—K16-0002, 15—K16-0004, 16—K16-0007, 17—K16-0018, 18—K16-0143.

Figure 1 shows fragments of electrophoregrams for the analysis of the *Sr2* (Figure 1a), *Sr31* (Figure 1b) and *Sr38* (Figure 1c) genes, respectively.

Samples 2, 5, 8, 12, 15, 16 and 17 have a diagnostic fragment of the functional allele (120 bp) of the *Sr2* gene (Figure 1a). Samples 1, 2, 5, 7, 8, and 10 in Figure 1b have a diagnostic fragment of the functional allele (207 bp) of the *Sr31* gene. Samples 3, 8, 10, 11, 12 and 18 (Figure 1c) have a diagnostic fragment of the functional allele of the *Sr38* gene. The remaining samples do not have the diagnostic fragments *Sr2*, *Sr31* and *Sr38*. Only sample 11 from Figure 1a is heterozygous.

**Figure 2:**
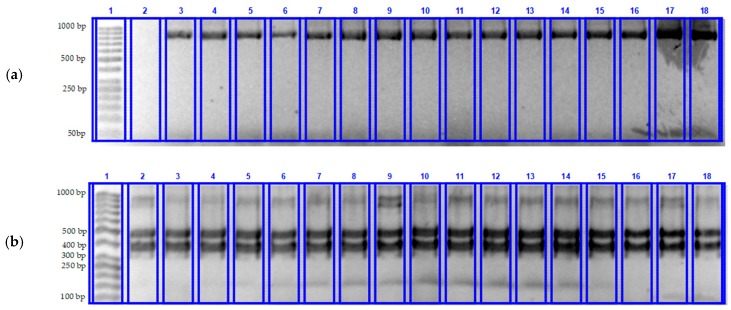
Determination of *Sr44* gene in winter wheat samples on agarose gel: (**a**)—before restriction: 1—Thermo Scientific GeneRuler 50 + bp molecular weight marker (50–1000 bp), 2—H_2_O deionized (negative control of experience), 3—K17-0350, 4—K17-0354, 5—K17-0357, 6—K17-0361, 7—K17-0372, 8—K17-0378, 9—K17-0393, 10—K17-0397, 11—K17-0411, 12—K17-0425, 13—K17-0426, 14—K17-0427, 15—K17-0428, 16—K17-0429, 17—K17-0430, 18—K17-0431; (**b**)—after restriction: 1—Thermo Scientific GeneRuler 50 + bp molecular weight marker (50–1000 bp), 2—Donskaya Polukarlikovaya (positive control of experience), 3—K17-0350, 4—K17-0354, 5—K17-0357, 6—K17-0361, 7—K17-0372, 8—K17-0378, 9—K17-0393, 10—K17-0397, 11—K17-0411, 12—K17-0425, 13—K17-0426, 14—K17-0427, 15—K17-0428, 16—K17-0429, 17—K17-0430, 18—K17-0431.

The separation of restriction products on a 2% agarose gel was used to identify the functional allele of the stem rust resistance *Sr44* gene. Figure 2 shows electrophoregram fragments of the analysis of the Sr44 gene before (Figure 2a) and after restriction (Figure 2b), respectively.

All samples in Figure 2a showed an amplicon measuring 874 bp. The amplification of the samples was successful. The samples with the identified amplicon of the Xbe404728 marker were cut using MSpI restriction endonuclease and separated on a 2% agarose gel (Figure 2b). Two separated fragments on the gel show the presence of the functional allele of the stem rust resistance *Sr44* gene.

The research data for each of the studied genes (*Sr2*, *Sr31*, *Sr38* and *Sr44*) were reduced to a binary form, where ‘1’ is the presence of the stem rust resistance gene, and ‘0’ is its absence.

**Figure 3:**
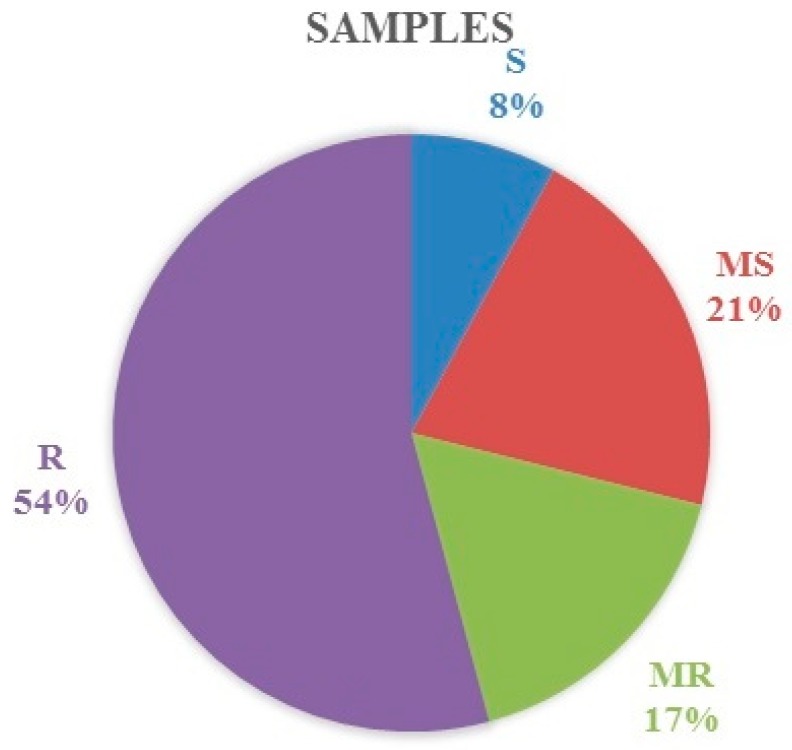
Distribution of winter wheat samples by groups of stem rust resistance.

Four groups of samples from the field tests, including susceptible (S), medium susceptible (MS), medium resistant (MR) and resistant (R), were identified (Figure 3).

In total, 49 samples (7.90%) were relevant to the susceptible group, 130 samples (20.97%) were relevant to the medium-susceptible group, 105 samples (16.94%) were relevant to the medium-resistant group, and 336 samples (54.19%) were relevant to the resistant group.

The final data table (field tests) is presented in Supplementary Table 1 (Table S1).

The differences between the groups were estimated by variance analysis. The results of the variance analysis, given in Table 1, show the statistical significance of the differences between groups.

There were also identified great differences between the average values in the groups estimated by the Duncan method (Table 2).

**Figure 4:**
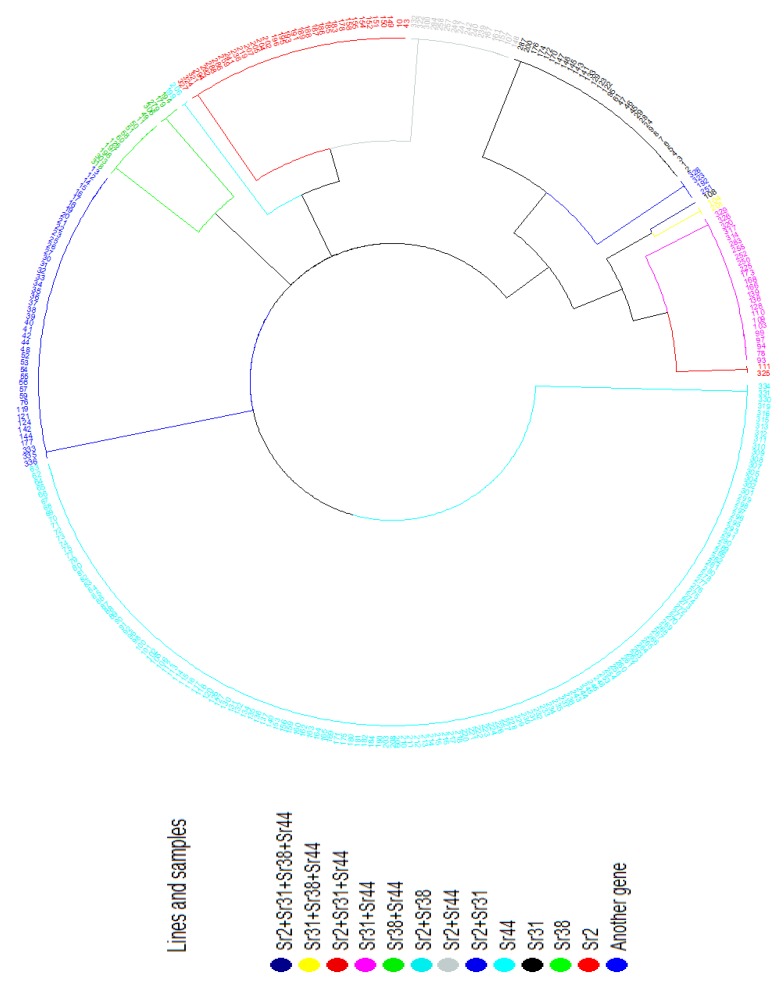
Cluster diagram of winter wheat samples of the group R.

Since stable samples are of the greatest interest in breeding, we have considered the R group in detail. This group was clustered by the binary method because our PCR analysis data were presented as a binary matrix. The samples were divided into 13 clusters (Figure 4).

The largest number of samples in the R group (156 samples (46.43%)) was included in the cluster with the presence of the single stem rust resistance gene *Sr44* (Table 3). The resistance of 10.42% in the samples (35 pcs.) was due to the presence of the *Sr2* gene.

The *Sr31* gene was represented in 32 samples (9.52%), and the *Sr38* gene was represented in 11 samples (3.27%).

Twenty-four samples (7.14%) were in Cluster 5 with a combination of the *Sr44* and *Sr31* stem rust resistance genes. Clusters 6 (*Sr44* and *Sr38* genes) and 9 (*Sr31* and *Sr2* genes) contained four samples (1.19%). Two samples (0.60%) belonged to Clusters 10, 11, and 13.

Cluster 15 included only 1 sample (0.30%) carrying all four stem rust resistance genes (K16-0147).

In the R group, there were no winter soft wheat samples identified belonging to Clusters 8, 12 and 14.

Cluster 16 had included 47 samples (13.99%), which indicates the presence of other effective resistance genes that we have not identified yet.

The cluster distribution of the samples was unbalanced. The relationship of the resistance of the samples with clusters was estimated by the Matthews correlation coefficient (Table 4) [21].

A weak positive correlation was identified for Clusters 2 and 5. The average negative correlation for Cluster 16 was determined (r = −0.56). The average positive correlation for Cluster 1 was identified (r = 0.36). Therefore, the presence of the *Sr44* gene affects the stability of winter soft wheat samples. The presence of another gene that we have not studied may have a partial effect on plant resistance. It can be either the stability gene already known in the world, or one not studied. Further research is needed.

We have identified the winter wheat samples with group stem rust resistance, having two or more effective resistance genes in various combinations (Table 5).

In total, 21 samples possessed group stem rust resistance. Most of these samples were the lines developed in the FSBSI “ARC “Donskoy”. The sample № 111was the cultivar ‘Don 85’ (WIR58516). The sample № 148 was the cultivar ‘Voyazh ‘. These varieties were developed in our Center and are actively used in breeding work. The varieties’ progeny may also have stem rust resistance genes *Sr2*, *Sr44* and *Sr31*.

## 3. Discussion

The *Sr31* gene has lost its stem rust resistance but is still a valuable resource. The gene is in translocation with resistance genes to other diseases. According to Yu (Yu et al.) [16], three QTLs found in the 1BS chromosome, which is homologous to 1RS, may be due to the residual effect of *Sr31*, or another gene obtained from rye translocation. According to Miroshnichenko [15], the *Sr31* gene keeps maintaining its effectiveness in Southern Russia, and the *Sr38* gene is low level effective, which has been proven by our results, as 32 winter wheat samples of the R group (9.52%) had the *Sr31* gene and 11 samples (3.27%) had the *Sr38* gene.

The stem rust resistance gene *Sr2* was recommended by Haile and Roder [22] for use in breeding programs in combination with other stem rust resistance genes. Rutkoski et al. have found that the locus of adult plant resistance, which includes the *Sr2* gene, plays an important role in the studied germplasm CIMMYT, and the use of genotypes as fixed effects in genomic breeding can give a good projection [23]. In our study, we identified 35 samples (10.42%) of winter soft wheat with this gene. These samples can be used as a testing group using genomic selection.

The *Sr44* gene is effective in Russia and in the world [24,25]. According to Baranova et al. [26] the gene is available in the cultivar ‘Donskaya Polukarlikovaya’, which is included in the breeding background of the studied lines. Since the *Sr44* gene has been identified in these lines, the cultivar ‘Donskaya Polukarlikovaya’ is a valuable donor for breeding for stem rust resistance.

The results of our study have shown that the *Sr44* gene and its combinations with other effective genes play a significant role for the stem rust resistance of winter wheat samples cultivated in Southern Russia. The varieties ‘Don 85’ and ‘Voyazh’ with the *Sr2* and *Sr44* genes identified in the study can be used as resistance sources against stem rust Ug99 in the breeding programs. These lines differ in other valuable indicators. Using one of these sources should be sufficient to transmit the resistance genes *Sr2* and *Sr44*. The cultivar “Voyage”, having the genes *Sr2* and *Sr44* and not having the gene *Sr31* that is affected by Ug99, will be more useful for breeding work. This information can be used in future breeding work for stem rust resistance.

## 4. Materials and Methods

### 4.1. Plant Material

The objects of the study were 620 collection samples of winter soft wheat. These samples were obtained from the VIR (Federal Research Center N. I. Vavilov All-Russian Institute of Plant Genetic Resources) and other scientific institutions as a result of the exchange of breeding material. Some of the lines were created in our center by the method of crossing and selecting breeding material. The studied samples have different ecological and geographical origins. A total of 63.69% of samples were from Russia, 9.08% from Ukraine, 7.38% from Germany, 7.08% from the USA, 10.62% from European countries, 1.69% from China and 0.46% from Kazakhstan. 

In total, 139 of the samples (22.42%) were winter soft wheat developed in the Federal State Budgetary Scientific Institution “Agricultural Research Centre “Donskoy” (FSBSI “ARC “Donskoy”).

The lines stored in VIR are available to any breeder upon request. Breeding lines created in our breeding center may be partially accessible.

### 4.2. Field Test

We studied all of the samples in an infectious background in the fields of the FSBSI “ARC “Donskoy” (46°50′42″ N, 40°18′30″ E) in 2016–2018. The most favorable conditions for the development of stem rust were in the year 2017.

The infection of the wheat varieties was carried out in the booting stage by dusting with a mixture of viable urediniospores of the stem rust race native to our region using the talcum powder method (flour used as a base). The spores were collected from infected plants against an infectious background. The spores were stored at room temperature, with a moisture content of 20–30%. [27]. The estimation of the stem rust response of the wheat varieties was carried out using the Peterson’s scoring scale [28]. The data obtained were divided into 4 groups according to the system for monitoring diseases, pests and weeds in cereal crops by the FAO: (The Food and Agriculture Organization of the United Nations) susceptible (S), medium susceptible (MS), medium resistant (MR) and resistant (R) [29].

### 4.3. DNA Extraction and Study

The genomic DNA was isolated from the leaves of 5–7-day-old seedlings using the modified CTAB protocol [30]. The quantity and quality of the DNA were assessed on an Implen NP80 spectrophotometer (Implen GmbH, Germany), and adjusted to 50 ng/μL. The primers used in PCR were synthesized by the Evrogen (Russia) and Syntol (Russia) companies (Table 6). PCR was performed in a volume of 25 μL.

We separated the amplification products on a 2% agarose or 8% polyacrylamide gel, and stained them with ethidium bromide fluorescent dye. GeneRuler 50 + bp (50–1000 bp) (Thermo Scientific, USA) and Evrogen 50 + bp (50–700 bp, Evrogen, Russia) were used as molecular weight markers. To identify the *Sr2* stem rust resistance gene, we used the molecular SSR marker Xgwm533, which is closely linked to this gene for use by authors [31,34,35,36]. The diagnostic Xgwm533 marker fragment was 120 bp. Identification of the *Sr31* gene was performed with the SCM9 marker, its diagnostic fragment was 207 bp. The VENTRIUP-LN2 marker was used to identify the *Sr38* gene. A diagnostic fragment size of 259 bp indicated the presence of this gene.

The samples successfully amplified with the Xbe404728 marker (*Sr44* gene) were used for restriction with endonuclease MSpI (NEB, USA). The composition of the reaction mixture was 10 μL PCR products, 5 μL separating buffer (1.5 μL 10 × Tango buffer; 3.4 μL H_2_O; 0.1 μL MSpI). The samples were incubated at 37 °C for 2 h and then evaluated on a 2% agarose gel. In the absence of the *Sr44* gene in the source material for analysis, the restriction did not occur. In the presence of the *Sr44* gene, 2 bands are observed.

### 4.4. Data Analysis

The winter soft wheat samples were assessed for stem rust resistance on the infectious background in the conditions of Southern Russia. After that, we studied the representation of stem rust resistance genes in our region (*Sr2*, *Sr31*, *Sr38* and *Sr44*) in 620 winter wheat samples by the PCR method.

The data analysis was carried out in the programming language R v. 3.5.1 [37] in the RStudio [38] program, with the help of specialized packages “agricolae” (estimation of differences between group mean values by the Duncan method) [39], “ape” (dendrogram analysis) [40], “cyclize” (circular visualization) [41], “dendextend” (visualizing and adjusting tree of hierarchical clustering by the binary method) [42], “DescTools” (ANOVA analysis) [43] and “dplyr” (data grouping and counting) [44].

## Figures and Tables

**Table 1:** Results of analysis of variance.

	Df	Sum Sq	Mean Sq	F Value	Pr(>F) ^a^
Group	3	647,279	215,760	4849	<2 × 10^−16^
Residuals	1236	54,993	44		

^a^ Level of significance

**Table 2:** Differences between average values of stem rust resistance groups.

Group	Diff	Lwr.ci	Upr.ci	*P*-Value ^a^
MS-MR	25.32601	24.11186	26.54015	<2 × 10^−16^
R-MR	−15.92262	−16.95718	−14.88805	<2 × 10^−16^
S-MR	56.40136	54.71582	58.08690	<2 × 10^−16^
R-MS	−41.24863	−42.25491	−40.24234	<2 × 10^−16^
S-MS	31.07535	29.52418	32.62652	<2 × 10^−16^
S-R	72.32398	70.78409	73.86387	<2 × 10^−16^

^a^ Level of significance

**Table 3:** Distribution of winter wheat samples in the clusters of the R group.

No of Cluster	Cluster	Number of Samples	The Percentage of Samples in the Group	No of Cluster	Cluster	Number of Samples	The Percentage of Samples in the Group
1	*Sr44*	156	46.43	9	*Sr31*_*Sr2*	4	1.19
2	*Sr31*	32	9.52	10	*Sr38*_*Sr2*	2	0.60
3	*Sr38*	11	3.27	11	*Sr44*_*Sr31*_*Sr38*	2	0.60
4	*Sr2*	35	10.42	12	*Sr44*_*Sr38*_*Sr2*	0	0
5	*Sr44*_*Sr31*	24	7.14	13	*Sr44*_*Sr31*_*Sr2*	2	0.60
6	*Sr44*_*Sr38*	4	1.19	14	*Sr31*_*Sr38*_*Sr2*	0	0
7	*Sr44*_*Sr2*	16	4.76	15	*Sr44*_*Sr31*_*Sr38*_*Sr2*	1	0.30
8	*Sr31*_*Sr38*	0	0	16	Another Gene	47	13.99

**Table 4:** The Matthews correlation coefficients.

Cluster	Correlation Coefficient	Cluster	Correlation Coefficient
1	0.36	9	0.07
2	0.14	10	0.05
3	−0.01	11	0.05
4	0.04	13	0.05
5	0.18	15	0.04
6	0.02	16	−0.56
7	0.06		

**Table 5:** Winter soft wheat samples with the group of stem rust resistance.

Genotype	Samples
*Sr44* + *Sr2*	107, 148, 157, 161, 167, 239, 240, 242, 247, 249, 257, 258, 284, 300, 322 and 332
*Sr44* +*Sr31* + *Sr38*	104 and 201
*Sr44* + *Sr31* + *Sr2*	111 and 325
*Sr44* + *Sr31* + *Sr38* + *Sr2*	108

**Table 6:** Markers linked to the resistance genes *Sr2*, *Sr31*, *Sr38* and *Sr44* with their forward and reverse primers.

Genes	Marker	Forward Primer	Reverse Primer	References
*Sr2*	Xgwm533	5′-GTTGCTTTAGGGGAAAAGCC	5′-AAGGCGAATCAAACGGAATA	[31]
*Sr31*	SCM9	5′-TGACAACCCCCTTTCCCTCGT	5′-TCATCGACGCTAAGGAGGACCC	[32]
*Sr38*	VENTRIUP-LN2	5′-AGGGGCTACTGACCAAGGCT	5′-TGCAGCTACAGCAGTATGTACACAAAA	[33]
*Sr44*	Xbe404728	5′-GGTGGTGCCTGTCAAGATT	5′-TTGATGGATCCTGGCTTAGG	[20]

## Supplementary Materials

The following is available online at https://www.mdpi.com/2223-7747/8/12/559/s1, Table S1: Stem rust resistance data in winter wheat samples.
